# Inhibition of Oxidative Phosphorylation Reverses Bone Marrow Hypoxia Visualized in Imageable Syngeneic B-ALL Mouse Model

**DOI:** 10.3389/fonc.2020.00991

**Published:** 2020-06-30

**Authors:** Mateusz Rytelewski, Karine Harutyunyan, Natalia Baran, Saradhi Mallampati, M. Anna Zal, Antonio Cavazos, Jason M. Butler, Sergej Konoplev, Mirna El Khatib, Shane Plunkett, Joseph R. Marszalek, Michael Andreeff, Tomasz Zal, Marina Konopleva

**Affiliations:** ^1^Department of Immunology, The University of Texas MD Anderson Cancer Center, Houston, TX, United States; ^2^Department of Leukemia, The University of Texas MD Anderson Cancer Center, Houston, TX, United States; ^3^Translational Molecular Pathology, The University of Texas MD Anderson Cancer Center, Houston, TX, United States; ^4^Weill Cornell Medicine, Medical School of Biological Sciences, Center for Discovery and Innovation, Hackensack University Medical Center, Nutley, NJ, United States; ^5^Department of Hematopathology, The University of Texas MD Anderson Cancer Center, Houston, TX, United States; ^6^Department of Biochemistry and Biophysics, The University of Pennsylvania, Philadelphia, PA, United States; ^7^TRACTION, The University of Texas MD Anderson Cancer Center, Houston, TX, United States

**Keywords:** hypoxia, oxidative phosphorylation, leukemia, oxygen, vascularity, acute lymphobastic leukemia, bone marrow

## Abstract

Abnormally low level of interstitial oxygen, or hypoxia, is a hallmark of tumor microenvironment and a known promoter of cancer chemoresistance. Inside a solid tumor mass, the hypoxia stems largely from inadequate supply of oxygenated blood through sparse or misshapen tumor vasculature whilst oxygen utilization rates are low in typical tumor's glycolytic metabolism. In acute leukemias, however, markers of intracellular hypoxia such as increased pimonidazole adduct staining and HIF-1α stabilization are observed in advanced leukemic bone marrows (BM) despite an increase in BM vasculogenesis. We utilized intravital fast scanning two-photon phosphorescence lifetime imaging microscopy (FaST-PLIM) in a BCR-ABL B-ALL mouse model to image the extracellular oxygen concentrations (pO_2_) in leukemic BM, and we related the extracellular oxygen levels to intracellular hypoxia, vascular markers and local leukemia burden. We observed a transient increase in BM pO_2_ in initial disease stages with intermediate leukemia BM burden, which correlated with an expansion of blood-carrying vascular network in the BM. Yet, we also observed increased formation of intracellular pimonidazole adducts in leukemic BM at the same time. This intermediate stage was followed by a significant decrease of extracellular pO_2_ and further increase of intracellular hypoxia as leukemia cellularity overwhelmed BM in disease end-stage. Remarkably, treatment of leukemic mice with IACS-010759, a pharmacological inhibitor of mitochondrial Complex I, substantially increased pO_2_ in the BM with advanced B-ALL, and it alleviated intracellular hypoxia reported by pimonidazole staining. High rates of oxygen consumption by B-ALL cells were confirmed by Seahorse assay including in *ex vivo* cells. Our results suggest that B-ALL expansion in BM is associated with intense oxidative phosphorylation (OxPhos) leading to the onset of metabolic BM hypoxia despite increased BM vascularization. Targeting mitochondrial respiration may be a novel approach to counteract BM hypoxia in B-ALL and, possibly, tumor hypoxia in other OxPhos-reliant malignancies.

## Introduction

Complex interactions between cancer cells and the tumor microenvironment lead to development of tumor hypoxia, which promotes cancer cell survival and treatment resistance. The hypoxia of a solid tumor core is thought to result largely from a local insufficiency of oxygenated blood supply rather than from oxygen utilization, which should be low in typically glycolytic-type tumor metabolism. B-cell acute lymphocytic leukemia (B-ALL) is an aggressive lymphoproliferative bone marrow (BM) malignancy that is commonly initiated by the Philadelphia chromosome p190 BCR-ABL translocation (1). Our group and others have reported that the BM microenvironment in leukemia is hypoxic with associated stabilization of hypoxia-inducible factor 1 alpha (HIF-1α) in leukemic cells (2–6). We previously found large areas of hypoxia, as detected by pimonidazole adduct staining, in the BM of patients with advanced stage refractory acute leukemia (AML and ALL) and in a murine ALL model (7, 8). Our studies identified that HIF-1α stabilization in stromal cells of the microenvironment facilitates leukemia homing and progression (9). Thus, BM oxygen levels are an important facet of the microenvironment and likely to influence leukemic biology and response to treatment (10–14). However, whether the origin of hypoxia onset in the BM with evolving acute leukemia is related to blood supply restriction or oxygen metabolism remains unknown.

Two specialized niches of the BM (the vascular and endosteal niches) are defined by their relative proximity to the local vasculature and bone surface/osteoblastic areas and play distinct roles in oxygen supply and normal BM physiology. Initial studies of the normal BM microenvironment suggested that the endosteum is poorly vascularized compared to the perivascular niches located deep within the BM cavity, and enriched with hematopoietic stem cells (HSC) exhibiting signs of hypoxia (15, 16). Those data and *in silico* modeling of hypoxic BM gradients (17, 18) initially led to an assumption that the hypoxia near endosteal niches is a consequence of low perfusion; however, direct *in vivo* O_2_ measurements in the mouse BM demonstrated that intravascular O_2_ levels were similar for endosteal and sinusoidal vessels (2.7 and 2.9%, respectively) (19). It was then shown that hypoxic HSCs are localized in the BM independent of their proximity to vascular or endosteal niches, and this led to the postulation that the inherently hypoxic nature of HSCs is a consequence of metabolism and O_2_ consumption by these cells, rather than anatomical location and perfusion (20).

In leukemias, leukemic stem cell (LSC) proliferation and chemoresistance is thought to be supported by the BM vascular niche, which consists mostly of sinusoidal endothelial cells and pericytes (21, 22). The intracellular formation of hypoxia-indicative pimonidazole adducts, and associated stabilization of HIF-1α in leukemia cells, could result from local oxygen depletion due to high rates of leukemic cell proliferation (6, 21). HIF-1α is a master transcriptional activator of angiogenic responses by inducing the expression of key factors including vascular endothelial growth factor A (VEGF-A), chemokine CXCL12, angiopoietin-2 (ANGPT2), placental growth factor (PGF), and platelet-derived growth factor (PDGF)-B (23). Indeed, there have been numerous reports of increased vasculogenesis in the setting of acute leukemia and myeloma, and more recent detailed imaging studies have demonstrated distinct vascular networks in normal and leukemic BM (24–27). Leukemogenesis induced not only hypoxia and BM angiogenesis, but also negatively altered the vessel integrity by inducing persistent vascular leakiness and increased NO levels (28).

We hypothesized that the hypoxia observed in leukemic BM is created by increased oxygen consumption driven by the metabolic demands of ALL cells, rather than a lack of blood supply to the BM cavities, and that this may represent a vulnerability for therapeutic exploitation. To address this question, we utilized intravital fast scanning two-photon phosphorescence lifetime imaging microscopy (FaST-PLIM) to image B-ALL cells and oxygen concentrations in the BM, in addition to pimonidazole staining to identify intracellular hypoxia, in the context of a transplantable BCR/ABL B-ALL mouse model (19, 29). We found that leukemia cells stained positive for pimonidazole despite increased vascularization of the BM and ample extracellular oxygen supply in the microenvironment at early stages of leukemia. Oxygen in the microenvironment dropped to sub-physiological levels and pimonidazole staining increased further in advanced disease stages. Inhibiting mitochondrial respiration with complex I inhibitor IACS-010759 reduced leukemia metabolic rates, abolished intracellular hypoxia, and normalized BM oxygen levels in advanced leukemia. This suggests that B-ALL expansion is sustained in large part by high rates of oxidative phosphorylation (OxPhos), and that targeting mitochondrial respiration with inhibitors of OxPhos may be a novel therapeutic approach to counteract B-ALL-associated BM hypoxia pathology.

## Results

### A Novel, Transplantable, and Imageable Mouse B-ALL Model Recapitulates Hallmarks of Human Disease

**Figure 1:**
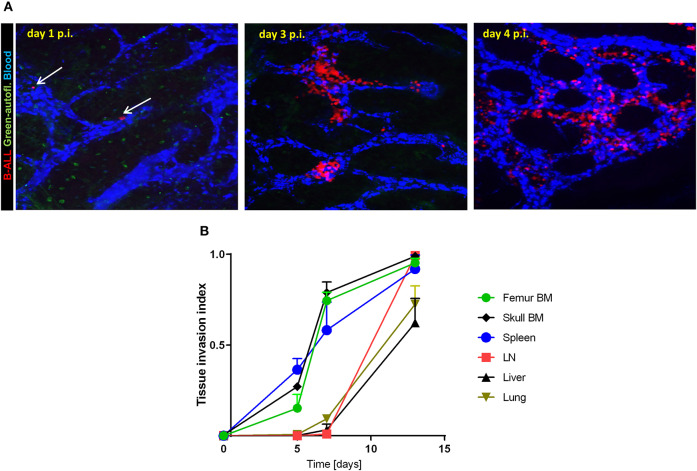
B-ALL engraftment and progression in immunocompetent recipient mice. **(A)** 2-photon intravital images of the calvarial bone marrow on day 1, 3, and 4 post-injection (p.i.) of 2.5 × 10^5^ B-ALL in C57BL6 mice. The blood was visualized using i.v. injection of BSA-AF647. The white arrows point to individual leukemia cells on day 1 p.i. **(B)** Leukemia tissue invasion index at different time points post-injection. B-ALL cells were injected in C57BL6 mice then tissues were collected and imaged (3 mice per time point day 5, 7, and 13 p.i.).

We generated a transplantable, imageable leukemia model by retrovirally transducing C57BL6-Ai14/CD19cre murine BM cells that express red fluorescing tdTomato with the p190-Bcr/Abl oncogene tagged with mCherry (30). The p190-BCR-ABL tdTomato/mCherry cells caused lethal leukemia in irradiated C57BL6 immunocompetent mice, manifested by infiltration of the spleen and bone marrow (Supplemental Figure 1). The immune-phenotype confirmed B-lymphoid origin of leukemic blasts (B220^+^, CD19^+^, CD4^−^, CD8^−^, CD11b^−^, not shown). Time-course imaging of intact calvarial and thinned femoral bones by multiphoton intravital microscopy (MP-IVM) demonstrated B-ALL cells lodged in BM on day 1 post-injection (Figure 1A, Supplemental Video 1). This was followed by rapid expansion of leukemia cells predominantly within the BM sinusoidal spaces, which were identified based on the flow of retro-orbitally injected BSA-AF647 fluorescent dye (Supplemental Videos 2, 3). Over the following 14 days, B-ALL cells populated the bone and spleen, followed by the lymph nodes, liver and lungs (Figure 1B). Prior to death, typically before day 21, mice exhibited rise in peripheral blood blasts and weight loss, and subsets of mice developed hind leg paralysis consistent with CNS involvement (not shown), reflecting the pathology of human B-ALL. Based on the skull BM tissue invasion index, defined as the average content of leukemia cell image surface in the BM cavity image surface, and taking into account the time since cancer cell infusion and animal symptoms, we qualitatively distinguished three disease stages in this model, namely *early leukemia* (BM invasion index ~ <10%, asymptomatic, days 1–4), *intermediate leukemia* (BM invasion index 10–60%, asymptomatic, days 5–12), and *advanced leukemia* (BM invasion index >60%, symptomatic, days 13–21).

**Figure 2:**
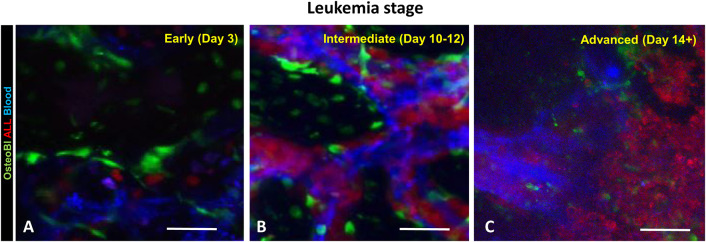
B-ALL cells localize and expand in the close proximity of blood vessels and osteoblasts (OB). The OB-GFP mice were infused iv with B-ALL-tdTomato cells followed by intravital 2-photon microscopy. **(A)** day 3 p.i.; **(B)** day 9 p.i.; **(C)** day 14 p.i. Red: B-ALL; blue: blood; green: GFP. Scale bars represent 100 μm.

To determine B-ALL cell localization and dynamics in the context of BM osteoblastic and vascular niches, we infused fluorescent B-ALL cells into transgenic mice expressing green fluorescent protein (GFP) in osteoblasts and osteocytes. As previously, the vascular niche was highlighted by intravenous fluorescent dextran and the bone was visualized by collagen second harmonic generation. The time course experiment (Figure 2) in these 2.3ColGFP/C57BL6 (OB-GFP) recipient mice demonstrated that the rapid expansion of leukemia cells within the sinusoidal spaces often occurred in the close proximity to osteoblasts (Figures 2A–C and Supplemental Video 4). Interestingly, while most leukemia cells were immotile, some leukemia cells exhibited amoeboid motility in the BM. As leukemia cellularity increased over the subsequent days, numerous leukemia cells became tightly packed throughout enlarged BM cavities such that most were located near the vasculature and some near the osteoblasts lining the bone. The cells were largely immotile except of those seen passing through the vascular bed with the blood flow (Supplemental Video 5). We observed loss of osteoblast numbers in the later stages of mouse leukemia, consistent with previously published reports (31, 32). This is consistent with invasion of both vascular and osteoblastic components of BM microenvironment.

### Dynamics of Oxygen in Leukemic Mice

**Figure 3:**
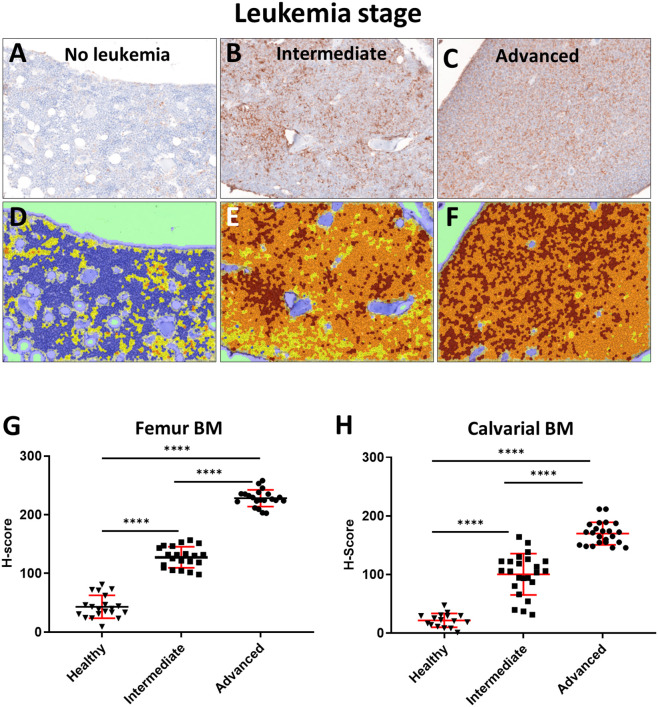
Evaluation of BM oxygenation using pimonidazole (Pimo) IHC. Mice were euthanized on specified day after injecting the leukemia cells and 3 h after ip injection of pimonidazole (3 mice per group). **(A–C**) Images of pimonidazole IHC in representative histological sections of formalin fixed paraffin embedded femurs (FFPE, 200x magnification). **(A)** C57BL6 mouse without leukemia. **(B)** Intermediate stage of leukemia (day 10+ p.i.). **(C)** Advanced stage of leukemia (day 14+ p.i.). **(D–F**) Corresponding IHC intensity and cell/tissue segmentation. Brown +3: high intensity of pimonidazole staining; Orange +2: medium intensity of pimonidazole staining; Yellow +1: low intensity of pimonidazole staining; Blue 0: negative/no intensity of pimonidazole staining. **(G)** Semi quantitative analysis (Histo-score, H-score) of the intensity of pimonidazole IHC in FFPE femur sections of leukemic and normal mice (Healthy): intermediate (Intermediate) and advanced (Advanced) stages of disease progression (*n* = 3 mice per time point). **(H)** H-score analysis of the pimonidazole signal intensity in FFPE skull sections in different stages of disease progression (3 mice per time point). ****p < 0.0001.

We have previously reported hypoxia in the BM of leukemic mice based on pimonidazole staining, which detects cells experiencing intracellular hypoxia (pO_2_ <10 mmHg) (33, 34). In the current study, we analyzed the time-course of pimonidazole-reported intracellular hypoxia in femur sections of mice sacrificed at specific times post ALL implantation: day 3 (*early leukemia*), day 10–12 (*intermediate leukemia*) and day 14+ (*advanced leukemia*) (Figure 3 and Supplemental Figure 2). No significant increase in pimonidazole binding was seen in early leukemia, with the overall pattern similar to that in the healthy control mice, indicating infrequent pockets of BM hypoxia consistent with prior reports (Supplemental Figure 2) (35). Longitudinal assessment of pimonidazole staining showed progressive development of intracellular hypoxia starting from day 10, reflecting high leukemic burden in the BM. We observed a statistically significant increase in Histo-score for pimonidazole IHC intensity on day 10–12 (*intermediate leukemia*) and day 14+ (*advanced leukemia*) in femur and calvarial BM *p* < 0.0001 (Figure 3).

**Figure 4:**
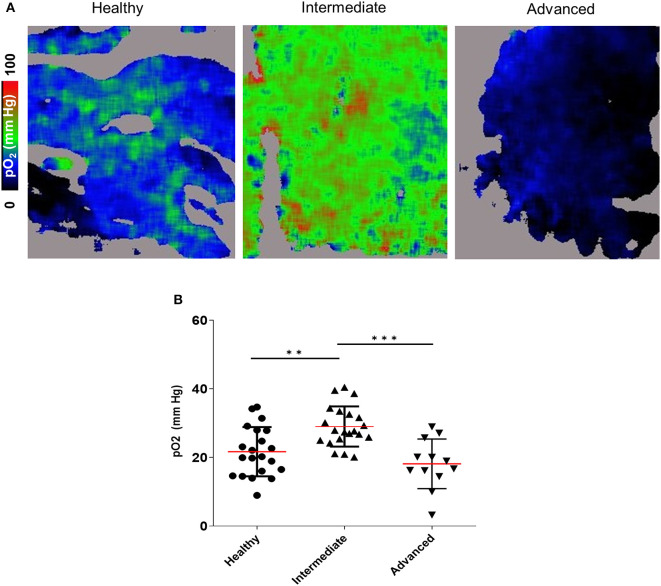
2-photon PLIM imaging of oxygen in the calvarial BM of mice with and without B-ALL. **(A)** Oxygen images showing pO_2_ levels represented in the color scale. **(B)** Mean pO_2_ levels. **p < 0.01, ***p < 0.001.

The observed intracellular hypoxia detected by pimonidazole adduct staining could reflect either inadequate oxygen supply to the BM extracellular spaces, or high rates of intracellular oxygen consumption (or both). To directly quantify the extracellular oxygen tension [pO_2_] in the BM microenvironment (as opposed to intracellular pimonidazole adduct staining), we utilized an imaging technique that enables high resolution contextual visualization of extracellular molecular oxygen based on probe phosphorescence lifetime along with local microenvironment structure and cellular composition based on multi-reporter fluorescence (36). Using this technique, termed FaST-PLIM (fast-scanning two-photon phosphorescence lifetime imaging microscopy), we performed time course imaging of the calvarial BM oxygenation at various leukemia stages. Remarkably, we observed a transient increase in the average BM extracellular pO_2_ at the intermediate stage of disease. This was followed by a statistically significant drop in BM pO_2_ at the advanced leukemia stage (Figure 4). These results demonstrate that leukemic cells in the BM with intermediate leukemia burden may experience a degree of intracellular hypoxia despite the presence of normal or supra-physiological extracellular oxygen levels. Nonetheless, as the cellular density of leukemia in the BM increased during advanced disease, the average extracellular oxygen levels dropped below the levels seen in normal BM and locally to hypoxic levels. Unlike the apparent disconnect between the extracellular and intracellular BM oxygen status in intermediate leukemia, in advanced leukemia the deepening intracellular hypoxia (as reported by pimonidazole staining) was consistent with widespread low levels of extracellular pO_2_ in leukemic BM.

### BM Angiogenesis and Vascular Network Disorganization During Leukemia Expansion

**Figure 5:**
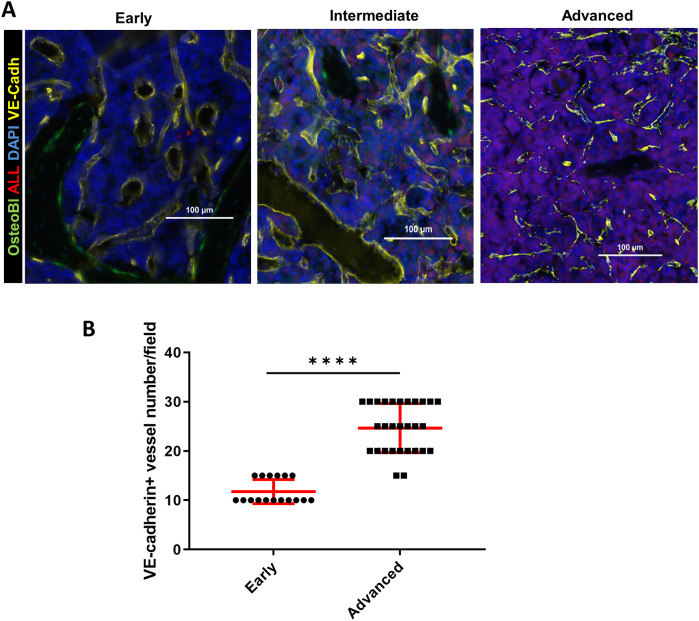
BM immunofluorescence microscopy of the vascular niche in leukemic OB-GFP mice at consecutive stages of leukemia progression. **(A)** Representative immunofluorescence images of frozen femur sections. Red: B-ALL (tdTomato fluorescence); yellow: VE-cadherin-Alexa647; green: osteoblasts (GFP); blue: DNA (DAPI) (representative of 3 mice per group). The early, intermediate and advanced stages of leukemia are defined in Results. (**B**) Quantification of VE-cadherin+ blood vessels in the bone marrow of mice with early vs. advanced B-ALL. ****p < 0.0001.

**Figure 6:**
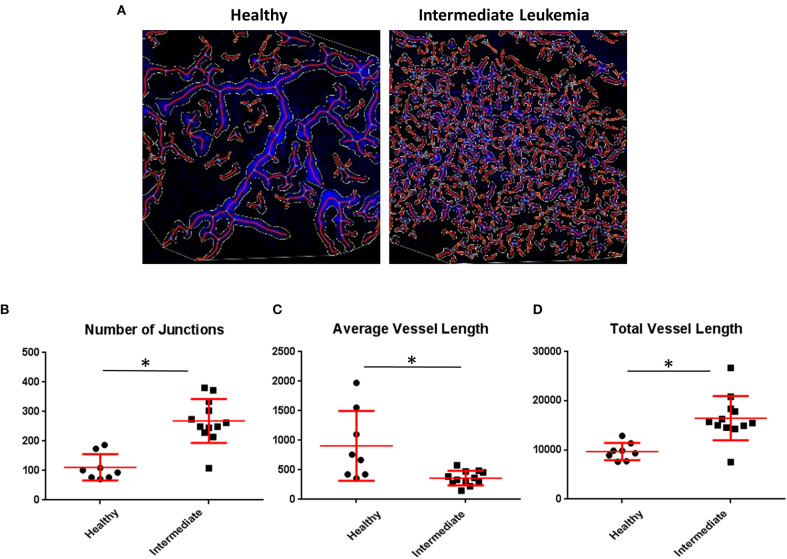
Quantitative imaging of BM vasculature. **(A)** Representative 2-photon intravital images were maximum intensity projected and evaluated using AngioTool and the output was overlaid. **(B)** AngioTool numbers of junctions; (**C**) AngioTool average vessels length; **(D)** AngioTool total vessels length. *Indicates Student *t*-test *p* < 0.05.

Given that we identified an increase in intracellular hypoxia staining (pimonidazole) in the BM of mice with intermediate leukemia despite the presence of ample extracellular oxygen (FaST-PLIM), we suspected that this apparent disconnect between the extracellular and intracellular oxygen status reflects a dynamic interplay of two evolving factors: the rate of oxygenated blood supply to BM and the local rates of oxygen consumption by B-ALL cells, which are becoming a major component of BM cellularity. We evaluated the status of femur BM vasculature during leukemia progression by immunofluorescence staining of blood vessel walls with intravenously injected Alexa-Fluor-647-conjugated VE-cadherin antibody (37) and in calvaria BM by intravital 2-photon imaging of dextran-TRITC-labeled blood flow. Femur VE-cadherin staining (Figure 5A) showed that while blood vessels were was largely unchanged between the early and intermediate stages of ALL progression, vessel density increased 2.1-fold in advanced ALL, compared to that in early leukemia (Figure 5B). For 2-photon analysis of calvaria BM blood flow, we used AngioTool software (Figures 6A–D) to quantify the number of capillary blood vessel junctions, average vessel length, and total vessel length. We found that density of capillary vessel junctions in mice with advanced leukemia (day 14 p.i.) increased 2.5-fold compared to that in the healthy control mice (Figure 6B) and the respective average vessel length decreased 2.2-fold (Figure 6C). Reflecting the increased meshwork-like nature of the leukemic vessels, the total length of all the vasculature per FOV increased about 1.7-fold (Figure 6D).

### Inhibition of Oxidative Phosphorylation Decreases Hypoxia in the BM

**Figure 7:**
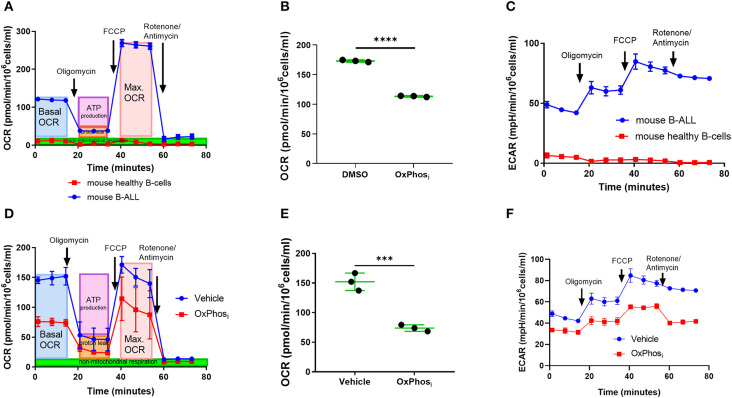
Inhibition of OxPhos decreases oxygen consumption by B-ALL cells. **(A)** Mitochondrial respiration (OCR) of *ex-vivo* B-ALL cells compared to mouse normal B cells. **(B)** Mitochondrial respiration of B-ALL cells treated with OxPhos_i_ (IACS-010759). **(C)** Extracellular acidification rate (ECAR) of B-ALL cells *ex-vivo* compared to mouse normal B cells. **(D)** Representative graph of mitochondrial respiration for BM cells from a mouse that received three doses of IACS-010759 (OxPhos_i_, 7.5 mg/kg) on day 12, 13, and 14 p.i. and control leukemic mouse BM cell measured by day 14 p.i. **(E**) Average basal mitochondrial respiration (BM cells) for 3 mice per group derived from mice treated with vehicle or IACS-010759 respectively, (day 14 p.i.). *p-value = 0.05. **(F)** Representative graph of extracellular acidification rate for BM cells from a mouse that received three doses of IACS-010759 (7.5 mg/kg) on day 12, 13, and 14 p.i. and control leukemic mouse BM cell measured on day 14 p.i. ***p < 0.001, ****p < 0.0001.

Thus, far, our findings suggested that leukemia progression is associated with a concomitant increase in BM micro-vessel density and higher supply of oxygen, before the metabolic demand for oxygen outpaces oxygen supply when BM cellularity becomes dominated by leukemia cells in advanced disease stages. Given the onset of intracellular and microenvironmental hypoxia at advanced-stage leukemia occurring despite a preceding increase of BM vascularization, we suspected that B-ALL blasts may be utilizing oxygen for oxidative phosphorylation (OxPhos) at much higher rates than normal BM cells. Indeed, Seahorse analysis of oxygen consumption rates (OCR) and extracellular acidification rates (ECAR) revealed that freshly isolated murine B-ALL cells avidly consumed oxygen compared with negligible rates of oxygen consumption by healthy murine BM-derived B-cells (Figures 7A–C). The high OCR in B-ALL was reduced by the inhibitor of complex I of the electron transport chain IACS-010759 (OxPhos_i_) (38), indicating a major role of OxPhos in oxygen metabolism in B-ALL (Figure 7B). Likewise, IACS-010759 effectively reduced OxPhos metabolism in leukemia cells *in vivo* as measured by reduced OCR and ECAR of freshly isolated leukemic BM after *in vivo* IACS-010759 treatment compared to that in the vehicle control at similar levels of tumor burden (Figures 7D–F).

**Figure 8:**
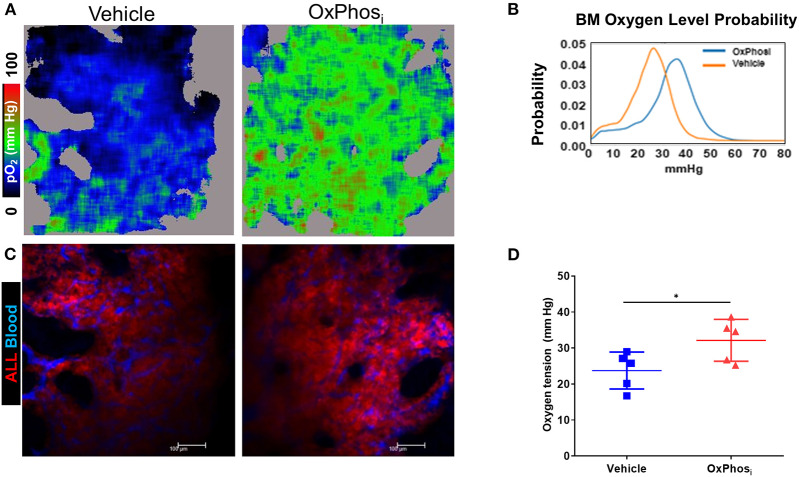
Complex I inhibition counteracts the hypoxia in the BM with advanced B-ALL. C57BL6 mice with B-ALL were treated with vehicle or Complex I inhibitor IACS-010759 (OxPhos_i_, 7.5 mg/kg for 3 consecutive days and 5 mg/kg for 3 more days). On indicated days, mice were infused i.v. with dextran-TRITC (blue) and PtP-C343 oxygen probe, anesthetized, and calvaria BM was imaged through intact skull by fast scanning two-photon phosphorescence lifetime imaging microscopy (FaST-PLIM) followed by quantification of pO_2_ levels. **(A)** Intravital oxygen images of skull BM on day 15 p.i. **(B)** Corresponding fluorescence images. **(C)** Local oxygen level probability distribution. **(D)** Mean oxygen tension in BM of B-ALL leukemic mice without treatment or with IACS-010759 treatment. *p-value < 0.05.

**Figure 9:**
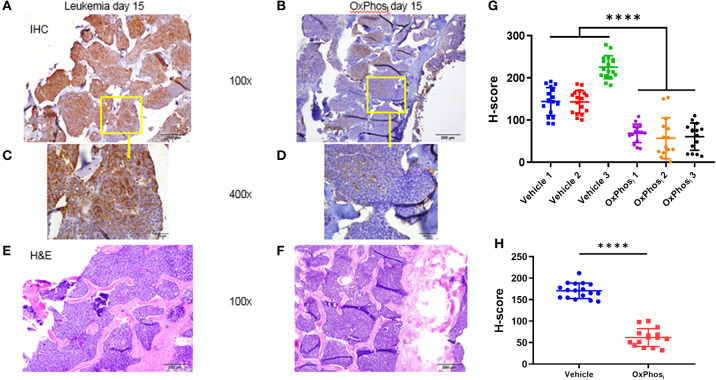
Evaluation of the effect of Complex I inhibitor (OxPhos_i_) on BM hypoxia using pimonidazole IHC. **(A,C)** representative IHC of skull section of untreated leukemic mouse and **(B,D)** IHC of skull section of IACS-010759 treated mouse; **(E,F)** H&E on corresponding adjacent sections. **(G)** Semi quantitative analysis of pimonidazole IHC intensity by Histo-score (H-score) for skull section of individual leukemic mice on day 15 p.i. **(H)** Average pimonidazole IHC intensity for 3 mice in each group day 15 p.i. ****p < 0.0001.

Based on these results, we sought to determine whether OxPhos inhibition can reverse BM hypoxia in advanced leukemia. For this purpose, we imaged both the extracellular oxygen levels *in vivo* and intracellular hypoxia status *ex vivo* in calvarial BM of mice with day 14 B-ALL within after infusing the mice with either vehicle or IACS-010759 Complex I inhibitor (Figures 8A–D, Supplemental Figure 3). The average BM pO_2_ in mice treated with IACS-010759 was about 35% higher than that in the vehicle treated mice (32.13 vs. 23.73 mmHg, respectively, *p* < 0.05, Figures 8C,D). Likewise, we observed a significant reduction in pimonidazole binding in mice following IACS-010759 administration (Figure 9). Histo-score analysis of the latter showed that the average score for leukemic mice skull pimonidazole signal dropped from 175 to 60 in IACS-010759-treated mice (Figures 9G,H). We observed a similar tendency in femur BM of IACS-010759-treated mice vs. leukemic mice treated with vehicle (Supplementary Figure 4).

## Discussion

In this study, we studied local oxygen status in mouse bone marrow during *in vivo* expansion of Ph^+^ B cell acute lymphoblastic leukemia by quantifying both intracellular oxygen signatures by pimonidazole staining and extracellular oxygen using two-photon phosphorescence oxygen microscopy. While confirming occurrence of BM hypoxia (<5% pO_2_) in advanced B-ALL disease, our results show that the state of BM oxygenation is dynamic during time course of leukemia expansion and driven by a high rate of mitochondrial respiration in leukemic cells factored by leukemic cell burden. By showing reversal of leukemic BM hypoxia in advanced B-ALL by mitochondrial complex I inhibitor IACS-010759, our results identify the metabolic process of oxygen consumption by mitochondrial respiration as a major contributor toward intratumoral hypoxia in B-ALL. The overall oxygen consumption by leukemic BM would increase with the local leukemia burden because the cell-intrinsic rate of oxygen consumption by mouse B-ALL cells was higher than that by normal BM B cells. In areas of high leukemia cell density, the local average rate of metabolic oxygen depletion may exceed the local rate of oxygenated blood delivery thereby resulting in a dynamic state of metabolic hypoxia that is amenable to reversal by OxPhos inhibition.

For the metabolic hypoxia to occur, such as we saw in advanced B-ALL, the local oxygen consumption rate had to exceed the rate of oxygen delivery by the blood flow. Interestingly, intravital oxygen imaging revealed a transient discordance between a gradual onset of intracellular hypoxia, as judged by *ex vivo* pimonidazole adduct staining, and transient elevation of the extracellular oxygen in developing disease, which was followed by decrease of extracellular oxygen tension including to hypoxic level in advanced disease stage. The apparent disconnect between pimonidazole reported intracellular hypoxia signature and the extracellular oxygen tension is consistent with prior reports showing no difference in pO_2_ levels measured by pulse oximeter in bone marrows freshly collected from primary AML patients compared to healthy bone marrow donors (39). As we and others (3, 40) have shown previously, AML and ALL patients' marrows, but not healthy BM, become profoundly hypoxic at the intracellular level, which leads to stabilization of main transcriptional regulator of hypoxia HIF-1α and its downstream targets such as CA9 in leukemia cells, despite ample oxygen delivery through the vasculature. In our experiments, the transient increase in extracellular oxygen levels was evident in intermediate leukemia burden and coincident with increased vascular density. It likely represented a stage whereby an increased average oxygen consumption rate was exceeded by the rate of oxygenated blood delivery due to ALL-driven BM vasculature expansion. At the same time, microscopic pockets of intracellular hypoxia could be developing in leukemia-dense areas and further away from nearest blood vessel. At individual cell level, high oxygen consumption rate inside a leukemic cell could deplete oxygen near the mitochondria allowing pimonidazole adducts to form there locally.

Our observation of increase in microvessel density during B-ALL expansion in BM is consistent with prior reports demonstrating that vascularity and angiogenic factors are increased in leukemias and may play a role in the leukemogenic process (24–27). Quantification of the percentages of perivascular Lin–CD48–CD41lo/–c-kit+ progenitor cells in contact with sinusoidal and non-sinusoidal endothelium (20) showed that c-kit+ progenitors display a hypoxic status regardless of their perivascular localization and distribution in the BM cavity. Our study has further shown that despite an overall increase in BM vascularization in response to the growth of leukemia, the average vessels' length is reduced with increased vessel cross-connectivity compared to normal mouse BM vessels. This may reflect poor functionality and leakiness of the misshapen vascular system under stressed conditions of leukemia microenvironment. Additional studies would be required to fully assess the functionality and permeability of diseased (or leukemic) compared to healthy BM vasculature. Our findings confirm profound microenvironment remodeling, including simultaneous increase of vascularity with decrease of vessel length, which might be a main contributor of niche dysfunctionality reported by others (28, 41). This might be a result of metabolically active endothelial cells involved in angiogenic process, requiring and consuming increased level of oxygen but being dysfunctional to supply nutrients and oxygen (41). While the expanded capillary network was effective in conducing the blood flow, as we saw using fluorescent dextran as blood marker, oxygen supply restriction could nonetheless occur despite manifestly increased BM vascularization, and adding to the metabolic hypoxia.

Our findings of BM hypoxia reduction by complex I inhibition with IACS-010759 clearly demonstrate the substantial metabolic origin of the BM intratumoral hypoxia. Resolution of measurable hypoxia upon arresting cellular respiration was likewise reported in multiple solid tumor models (38, 42, 43). The distinct oxidative metabolic requirements of leukemia (AML and recently CML) compared to normal hematopoietic stem cells (HSC) that predominantly utilize glycolysis for energy homeostasis, have been reported previously; and our work for the first time demonstrated a direct link to hypoxic responses. Pathological hypoxia is present in the majority of human solid cancers and is associated with increased tumor aggressiveness and therapeutic resistance, in part through activation of HIF-1α (3, 40, 44–46). While in solid tumors hypoxia is postulated to ensure as a result of reduced oxygen availability due to dysfunctional angiogenesis, recent findings indicate that heightened mitochondrial activity largely contributes to tumor hypoxia and HIF-1α stabilization; further, a series of known OxPhos inhibitors, including IACS-010759 used here, were initially developed as HIF-1α inhibitors (47–49). It remains to be seen, for example by overexpression of non-degradable HIF-1α, to what extent inhibition of hypoxic responses by mitochondrial inhibitors contributes to overall anti-tumor activity of these agents.

As shown by studies of solid tumors, one clinically adverse effect of intratumoral hypoxia is to promote cancer chemoresistance. Whether the hypoxia of leukemic BM acts similarly to promote chemoresistance in resident leukemia cells, and whether counteracting BM hypoxia would sensitize leukemia cells to chemotherapy, is only beginning to be explored. In this regard, the model tested does not reflect resistant disease, and additional studies are needed to evaluate the impact of BM hypoxia reduction on leukemia cell sensitivity to chemotherapeutic treatments. While we did not address the role of hypoxia in leukemogenesis and chemoresistance in current work, the reports from our group and others have demonstrated prevalence of hypoxia in human leukemic BM and negative prognostic impact of HIF-1α expression in newly diagnosed patients with pre-B-ALL (3); *in vitro* observation of hypoxia promoting chemoresistance in ALL (50, 51) and in AML (52–55); link between the poorly oxygenated niche, the hypoxia-induced glycolytic metabolism and chemoresistance in B-ALL and T-ALL (56, 57); and requirements for HIF1α signaling for leukemia stem cell maintenance in T-ALL (58). We have further demonstrated that residual leukemic bone marrow cells in a syngeneic AML model remain hypoxic after chemotherapy (59). Altogether these data support the pivotal role of hypoxia signaling in leukemias (60). Albeit our conclusions in the current study are limited to demonstrating the utility of complex I inhibition in alleviating the leukemic BM hypoxia, this finding may ultimately lead to important translational implications to be tested in subsequent studies. As an example, recent work by Farge et al. has demonstrated that preexisting and persisting, chemotherapy-resistant residual leukemia cells in AML PDX models exhibited an enriched high OxPhos gene signature and high pimonidazole positivity, and pharmacologic inhibition of OxPhos enhanced cytotoxic effects of AraC *in vitro* and *in vivo* (61).

In summary, our study demonstrates progressive alterations in the oxygenation of BM microenvironment during mouse B-ALL development and a pivotal role of leukemic cell mitochondrial respiration in the formation of leukemic BM hypoxia. We present new findings of development of hypoxia in intracellular level prior to oxygen tension decrease extracellularly. We also demonstrate efficacy of complex I inhibitor IACS-010759 to reverse leukemic BM hypoxia, which suggests a possibility of an additional previously unrecognized therapeutic benefit of this class of compounds. Further pre-clinical studies need to be conducted to elucidate therapeutic potential of using electron transport complex inhibitors or other types of OxPhos inhibitors to alleviate adverse effects of BM hypoxia in BM malignancies.

## Methods

### Mice: Breeding and Care

All animal experiments were performed under the approval of the Institutional Animal Care and Use Committee of the University of Texas MD Anderson Cancer Center. C57BL/6J mice (C57BL6, stock # 000664); B6.129P2(C)-Cd19tm1(cre)Cgn/J (CD19-Cre mice; stock #006785), B6;129S6-Gt(ROSA)26Sortm14(CAG-tdTomato)Hze/J (Ai14 mice; stock #007908), B6.Cg-Tg(Col1a1^*^2.3-GFP) 1Rowe/J mice (62) (2.3ColGFP mutant mouse strain; stock #013134) were obtained from the Jackson Laboratory. All mice were bred and housed in a specific pathogen-free animal facility at the University of Texas MD Anderson Cancer Center. Ai14 mice were mated to CD19-Cre mice to generate CD19-Cre/ Ai14 mice. Genotyping was performed by PCR using genomic DNA extracted from mouse tails according to Jackson laboratory protocols. 30% of B-cells (B220 positive) express tdTomato in CD19-Cre/ Ai14 mice (63). Ai14/CD19cre mouse bone marrow was used for experiments.

### Generation of Transplantable Imageable Mouse Model

We generated a transplantable, imageable B-ALL model by retrovirally transducing C57BL6-Ai14/CD19cre murine BM cells with the p190-Bcr/Abl oncogene tagged with mCherry (30). Thus, transduced bone marrow was infused into irradiated C57Bl6 mice resulting in development of leukemic disease and animal death within 28 days (Supplemental Figures 1A,B). BM cells of the primary recipient mouse were collected on day 21 post reconstitution with the transduced BM and the cells were cultured for 7 days. The outgrowing p190-BCR-ABL cells expressed high levels of tdTomato and low levels of mCherry, and were sorted out based on the fluorescence. Upon transplantation into secondary immunocompetent C56BL6 recipients, the cells (B220^+^ CD19^+^ CD4^−^ CD8^−^ CD11b^−^) triggered ALL development with animal life span of 14-16 days.

### Bone Marrow Cell Transduction and Establishment of p190 BCR-ABL Cells

Bone marrow cells from 4 weeks old CD19/Ai14 heterozygous female were isolated by using a flushing technique (64). Single cell suspension was then unreached for pro-B-Cell by using manual MACS columns and B220 beads [CD45R (B220) MicroBeads, cat # 130-049-501]. OP9 and pro-B cell co-culture was established as described before (30) and pro B cells were cultured for week in αMEM (alpha minimum essential medium (Cellgro) supplemented with 20% fetal bovine serum (FBS; ATCC), 2 mM glutamine (Gibco), 100 units/ml penicillin and 100 μg/ml streptomycin (Gibco) 50 μM β-mercaptoethanol (Sigma), and 10 ng/ml IL-7 (Miltenyi Biotec). The pro B cells were transduced by spinoculation with MIG-p190BCR-ABL-mCherry Retroviral supernatant in the presence of 5 μg/ml polybrene for 45 min at 450 g and 37°C with DMEM (Gibco) culture medium supplemented with 10% heat-inactivated FBS (Atlanta Biologicals), 100 units/ml penicillin, and 100 μg/ml streptomycin. Preparation of the viral vector and viruses were performed as it described (30). Transduced cells were then expanded and 1 million cells transplanted into sub-lethally irradiated (700 cGy) recipient mice by i.v. injection.

### Cell Growth and Flow Cytometry

BM cells were growing *in-vitro* in αMEM supplemented media (as described above). Upon transformation, B-ALL cells did not require IL-7 supplementation.

Flow cytometry was performed using Gallios 561 system (BD Biosciences). Flow sorting of antibody-stained cells was performed using an Astrios system (Beckman-Coulter). The sorted cells were determined to be of at least 98% purity on reanalysis using the Astrios system. The following antibodies were purchased from Tonbo Biosciences: allophycocyanin (APC)-conjugated anti-B220, fluorescein isothiocyanate (FITC)-conjugated anti-CD4, fluorescein isothiocyanate (FITC)-conjugated anti-CD8, fluorescein isothiocyanate (FITC)-conjugated anti-CD11b, (APC)-conjugated anti-CD19.

### Immunostaining of Bone Sections

Mice were injected by retro-orbital sinus with 100 μl/10 μg of Alexa Fluor 647-VE-cadherin (BV13) (37). After 10 min, bones from control and leukemic mice were fixed overnight in 4°C with 4% paraformaldehyde and were decalcified for 4 days in 10% EDTA (pH 7.2) at room temperature. Femurs were washed with PBS, pH 7.4, for 30 min, placed in 30% sucrose at 4°C overnight. Optimal cutting temperature compound OCT embedding and sectioning was performed by RNA *in-situ* Hybridization Core at Baylor College of Medicine. Frozen sections were post-fixed for 15 min with 4% paraformaldehyde and washed three times with PBS. Samples were mounted using Vectashield (Vector Laboratories). For assessment of bone marrow vasculature, slides were stained with DAPI (Molecular Probes, 1:5000) and all the cells was detected by fluorescence multispectral imaging system, Perkin Elmer Vectra 3 multispectral microscope. All specimens including unstained samples were imaged to identify and remove auto fluorescence from the images which substantially improves signal to noise measurements and intensity comparisons across fields.

### Metabolic Labeling and Treatments *in vivo*

IACS-010759 (OxPhos_i_) was administered by oral gavage at dose 7.5 g/kg or 5 mg/kg in 0.5% Methylcellulose solution. 10 mg/ml ketamine and 1 mg/ml xylazine cocktail was injected intraperitoneally (i.p.) at a dose of 10 μl/g of body weight to anesthetize mice before imaging. High molecular weight and 65–85 kDa TRITRC dextran, medium molecular weight (Sigma-Aldrich, Gillingham, (Dorset, UK) and BSA-AF647 (Invitrogen) for blood vessels visualization was injected right before imaging the bone marrow [10 μl of 1 mg/ml BSA-AF647 (Invitrogen) in 40 μl of PBS]. Pimonidazole probe PIMO was administered (i.p.) at 100 mg/kg. Three hours later, mice were euthanized and their bones were processed for IHC.

### Cell Oxygen Consumption Measurements *in vitro*

B-ALL cells were suspended in normal growth medium at a concentration of 3.0 × 10^6^ cells/ml and treated with Vehicle or IACS-010759 for 4 h, centrifuged and suspended in pre warmed (37°C) Seahorse medium (Seahorse XF basal medium supplemented with 2 mM Glutamine, 10 mM glucose, 1 mM pyruvate, pH 7.4). 175 μl of cell suspension containing 300,000 cells were seeded added to Seahorse 96-well plates pre coated with Cell-Tak. Plates were gently centrifuged at 1,000 g for 4 min. Seahorse analyses for cell lines and normal mouse B-cells were performed according to Seahorse Biosciences protocol for the Mito-Stress test. Basal OCR and ECAR analyses were performed using reagents from Seahorse Bioscience, as previously reported (65). Inhibitor concentrations were 1.5 μM for oligomycin, 1.0 μM for FCCP and 0.5 μM for antimycin or rotenone. All calculations were carried out by the Agilent Seahorse Mito Stress Generator after normalizing to cell number.

### Immunohistochemistry

Mouse femurs and skulls were harvested at specified time points and fixed by immersion in 4% PFA. Formalin-fixed, paraffin-embedded (FFPE) sections of bone was prepared after decalcification of all bone specimens. Sections were stained with H&E (x5000; Sigma-Aldrich) and analyzed by light microscopy (Olympus BX43). For pimonidazole IHC formalin-fixed, paraffin-embedded sections of bone were processed as described (66) using primary antibody—Rabbit IgG1 polyclonal anti-pimonidazole (Hypoxyprobe, Inc.) and secondary antibody Anti-Rabbit IgG (Goat), HRP-Labeled (Perkin Elmer). After DAB (3,3′-diaminobenzidine in chromogen/ Dako) visualization and hematoxylin (Dako) counterstaining coverslips were mounted with Fluoromount-G (Electron Microscope Science). Vectra 3 multispectral microscope captured IHC images were next quantified and scored by the inForm software (Perkin Elmer) and Histo-score standard analysis (H-score) were used to compare intensity of pimonidazole IHC in different samples. H-score method assigned a score of 0–300 to each sample, based on the percentage of cells stained at different intensities of IHC (67).

### Intravital Two-Photon Microscopy—Specimen Preparation

Mice were injected intravenously (i.v.) with 0.25 million B-ALL cells in 200 μl Hank's Balanced Salt Solution (HBSS) or PBS. At specified time points, mice were anesthetized using ketamine/xylazine cocktail injection (i.p.) followed by additional dosing every 20–30 min to maintain anesthesia. The scalp skin and underlying membrane were removed surgically to access the skull bone marrow for imaging. Following this procedure, the head was immobilized with a custom-made stereotactic holder on a heated motorized microscope stage maintained at 37°C throughout the entire imaging session. To visualize blood flow, the mice were infused with high molecular weight or 65–85 kDa TRITRC dextran (Sigma-Aldrich, Gillingham, Dorset, U.K.) and/or 10 μg BSA-AF647 (Invitrogen) in 0.1 ml PBS.

### Intravital Two-Photon Microscopy—Oxygen Probe and FaST-PLIM

Oxygen imaging was performed by fast scanning two-photon phosphorescence lifetime microscopy (FaST-PLIM) as described (36). Briefly, mice were infused i.v. with 40 μl of 1.7 mM solution of the PtP-C343 oxygen probe (68). Phosphorescence was captured using Leica SP5 microscopy laser scanner with a HyD detector and Becker & Hickl SP-120 system in a 256 × 256-pixel format at 10 Hz bi-directional line scanning. Average lifetimes were determined for each pixel using Becker & Hickl SPCI software followed by converting into oxygen tension (mmHg) values based on the PtP-C343 batch calibration. The fluorescence component data were captured in a 512 × 512-pixel format at 600 Hz bi-directional line scanning in four channels making use of two HyD and two PMT detectors. The fluorescence and oxygen images were combined, registered and further analyzed using Imaris (Bitplane, Inc.) and other software.

### Other Types of Image Analysis

Leukemia tissue invasion index was calculated based on image segmentation using Imaris software. Morphological parameters of the vasculature were quantified using AngioTool, a computational tool for quantitative analysis of vascular networks (69).

### Statistical Analysis

The student's *t*-test was used to evaluate the null hypothesis that there was no difference between two groups, and a *p*-value of < 0.05 was considered statistically significant a priori. ANOVA with Tukey multiple comparison test was used where the means of more than two groups were to be compared simultaneously. Unless otherwise noted, all calculations were carried out by the GraphPad Prism software (GraphPad) software.

## Data Availability Statement

All datasets generated for this study are included in the article/Supplementary Material.

## Ethics Statement

The animal study was reviewed and approved by Institutional Animal Care and Use Committee of the University of Texas MD Anderson Cancer Center.

## Author Contributions

All authors listed have made a substantial, direct and intellectual contribution to the work, and approved it for publication.

## Conflict of Interest

The authors declare that the research was conducted in the absence of any commercial or financial relationships that could be construed as a potential conflict of interest.
