# Supplementary material for: Inhibition of Oxidative Phosphorylation Reverses Bone Marrow Hypoxia Visualized in Imageable Syngeneic B-ALL Mouse Model
Source: Front Oncol. 2020 Jun 30;10:991. doi: 10.3389/fonc.2020.00991 (PMC7339962; doi:10.3389/fonc.2020.00991)
Supplement: Supplementary file 6 [file Data_Sheet_1.pdf]

Supplemental Figure 1

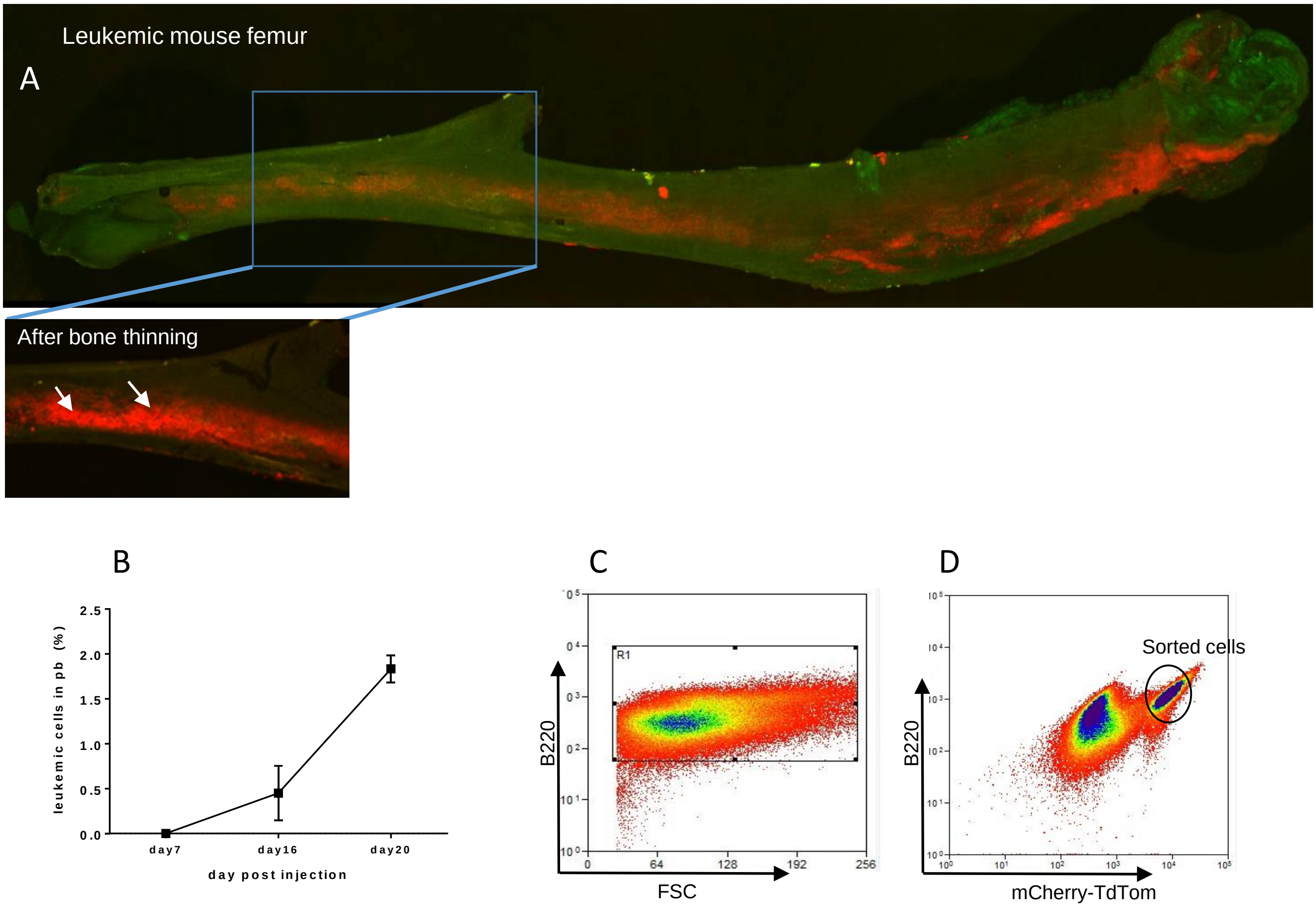

Supplemental Figure 1. Characterization of the p190 Bcr/Abl-driven mCherry-TdTomato B-ALL model in irradieated C57BL6 mice. 1 million primary tdTomato/ mCherry p190 Bcr/Abl cells were infused intravenously (i.v.) into irradiated (700cGy) C57BL6 mice (n=3). (A) Confocal images of a leukemic mouse femur on day 21 post injection (p.i.). The red color represents tdTomato fluorescence. The white arrows point to bright red leukemia cell locations. The green represents tissue autofluorescence. (B) Peripheral blood (pb) content of tdTomato/mCherry p190 Bcr/Abl cells in irradiated C57BL6 mice over time. Peripheral blood samples were evaluated by flow cytometry to measure the content of red fluorescing cells (n=3). (C and D) Flow sorting of B220/mCherry/ TdTomato positive cells. BM cells of the primary recipient mouse (day 21 p.i.) were collected and cultured in-vitro for 7 days then flow-sorted for TdTomato/mCherry and B220.

Supplemental Figure 2

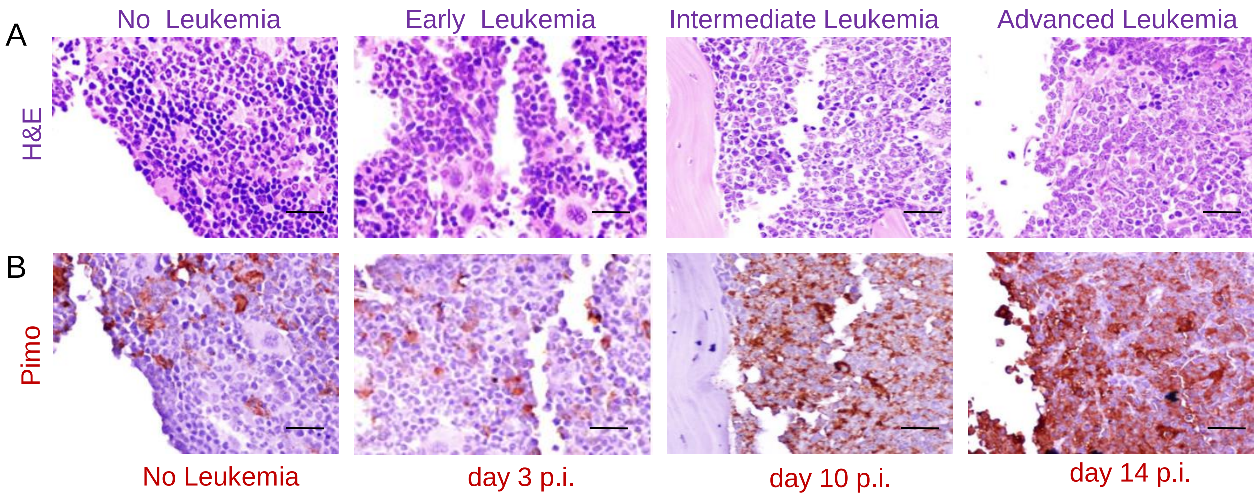

Supplemental Figure 2. Evaluation of bone marrow hypoxia by pimonidazole adduct (Pimo) immunohistochemistry (IHC). Femurs were dissected from mice euthanized on the specified days after leukemia implantation and 3 hours after intraperitoneal infusion of Pimo, embedded in paraffin, fixed in formalin, sectioned and stained. (3 mice per group). (A) H&E staining. (B) Pimo IHC on the corresponding adjacent sections. Scale bars represent 50 µm.

Supplemental Figure 3

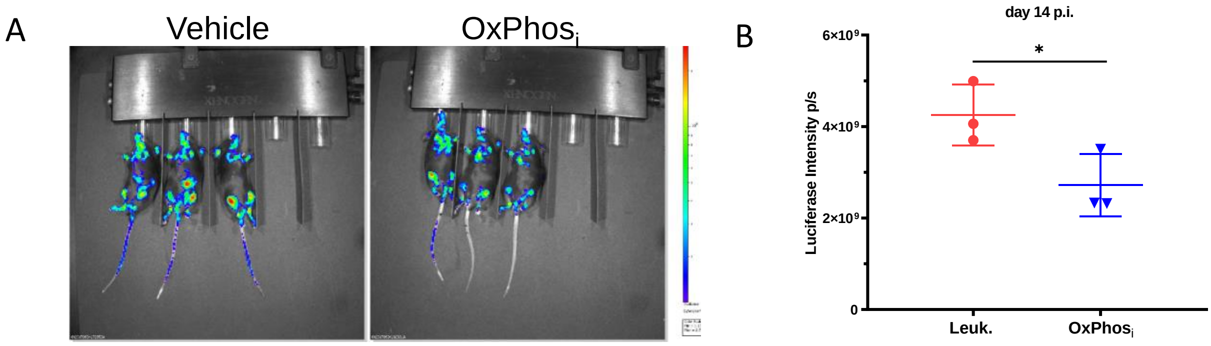

Supplemental Figure 3. In vivo bioluminescence imaging of leukemia burden. B-ALL-luciferase cells were transplanted into six C57BL6 mice and 3 mice were treated with OxPhos$_i$ (3 doses of 7.5 mg/kg on days 8, 9 and 10 p.i. and 3 doses of 5 mg/kg on days 13,14 and 15 p.i.). (A) D-luciferin bioluminescence images after the treatment. (B) Quantification of leukemia burden in terms of whole body bioluminescence intensity. The levels of leukemia in non-treated and drug treated mice were statistically significant. *p-$value$ < 0.05

Supplemental Figure 4

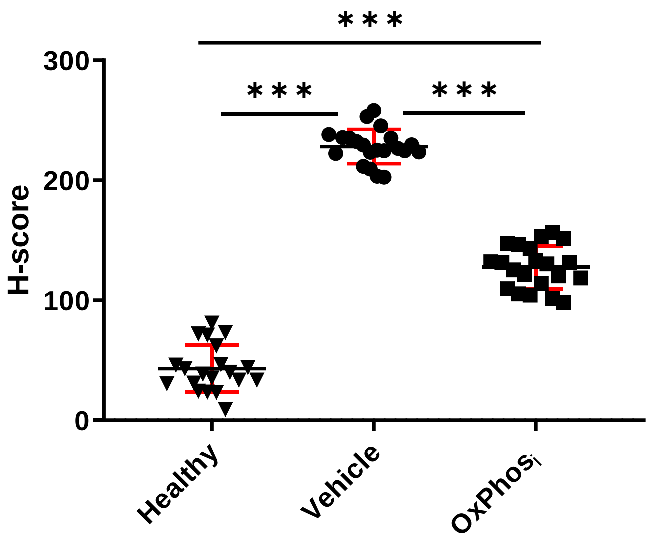

Supplemental Figure 4. H-score for pimonidazole IHC in femur BM. Mice with leukemia were randomized into two groups of which one was treated with 6 doses of OxPhos inhibitor. ****p-value* <0.001.

Supplementary Video Legends

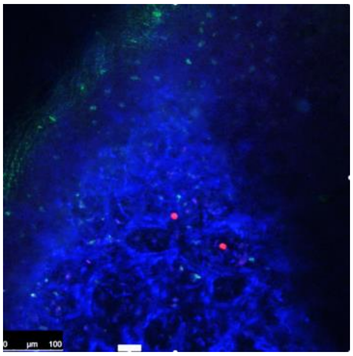

Supplemental Video 1. Two-photon microscopy travel through increasing depth of focus in live bone/bone marrow recorded one day after intravenous infusion of red-fluorescent B-ALL cells. The blue color is of the blood dye BSA-AF647. The green color is of autofluorescence.

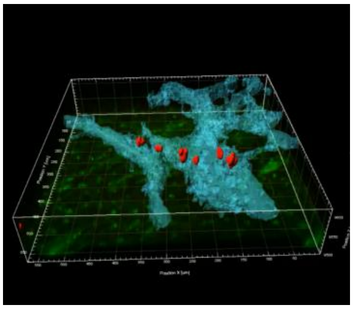

Supplemental Video 2. 3-D isosurface rendering of a z-stack of intravital two-photon microscopy images of a cluster of red-fluorescent B-ALL cells on post-infusion day 2, showing the proximity of leukemia cells to blood vessels. The blue or cyan color is of the blood stain BSA-AF647. The green color is of autofluorescence.

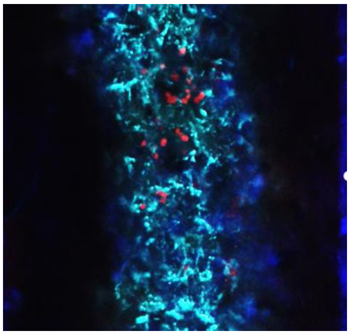

Supplemental Video 3. Two-photon microscopy travel through increasing depth of focus in fresh femur bone/bone marrow recorded on day 5 after intravenous infusion of red-fluorescent B-ALL cells. The cyan color is of the blood dye BSA-AF647. The blue color represents the second harmonic generation by bone collagen (SHG).

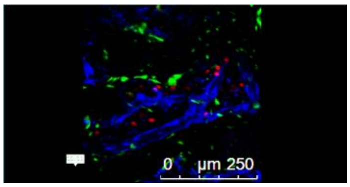

Supplemental Video 4. Intravital time-lapse two-photon microscopy recording inside of the calvaria bone marrow on day 3 after intravenous infusion of red-fluorescent B-ALL cells in OB-GFP mouse. The green color is of the osteoblasts expressing GFP. The blue color is of the blood dye BSA-AF647.

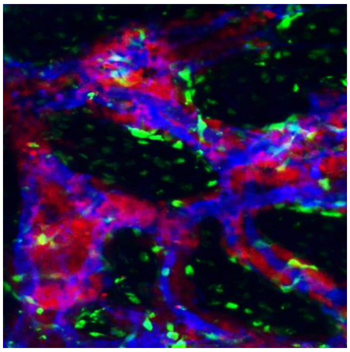

Supplemental Video 5. Intravital time-lapse two-photon microscopy recording inside of the calvaria bone marrow on day 9 after intravenous infusion of red-fluorescent B-ALL cells in OB-GFP mouse. The green color is of the osteoblasts expressing GFP. The blue color is of the blood dye BSA-AF647.
